# Validation of Commercial SARS-CoV-2 Immunoassays in a Nigerian Population

**DOI:** 10.1128/Spectrum.00680-21

**Published:** 2021-10-06

**Authors:** Fehintola Ige, Yohhei Hamada, Laura Steinhardt, Nnaemeka C. Iriemenam, Mabel Uwandu, Stacie Marta Greby, Maureen Aniedobe, Babatunde Lawal Salako, Molebogeng X. Rangaka, Ibrahim Abubakar, Rosemary Audu

**Affiliations:** a Center for Human Virology and Genomics, Microbiology Department, Nigerian Institute of Medical Research, Yaba, Lagos, Nigeria; b Institute for Global Health, University College London, London, United Kingdom; c Malaria Branch, Division of Parasitic Diseases and Malaria, Center for Global Health, Centers for Disease Control and Prevention, Atlanta, Georgia, USA; d Division of Global HIV and TB, Center for Global Health, Centers for Disease Control and Prevention, Abuja, Nigeria; e Clinical Diagnostic Laboratory, Nigerian Institute of Medical Research, Yaba, Lagos, Nigeria; f Nigerian Institute of Medical Research, Yaba, Lagos, Nigeria; Houston Methodist Hospital

**Keywords:** SARS-CoV-2, serology, validation, Nigeria, immunoassay, assay

## Abstract

Validated assays are essential for reliable serosurveys; however, most SARS-CoV-2 immunoassays have been validated using specimens from China, Europe, or U.S. populations. We evaluated the performance of five commercial SARS-CoV-2 immunoassays to inform their use in serosurveys in Nigeria. Four semiquantitative enzyme-linked immunosorbent assays (ELISAs) (Euroimmun anti-SARS-CoV-2 nucleocapsid protein [NCP] immunoglobulin G [IgG], Euroimmun spike SARS-CoV-2 IgG, Mologic Omega COVID-19 IgG, Bio-Rad Platelia SARS-CoV-2 Total Ab) and one chemiluminescent microparticle immunoassay (Abbott Architect SARS-CoV-2 IgG) were evaluated. We estimated the analytical performance characteristics using plasma from 100 SARS-CoV-2 PCR-positive patients from varied time points post-PCR confirmation and 100 prepandemic samples (50 HIV positive and 50 hepatitis B positive). The Bio-Rad assay failed the manufacturer-specified validation steps. The Euroimmun NCP, Euroimmun spike, and Mologic assays had sensitivities of 73.7%, 74.4%, and 76.9%, respectively, on samples taken 15 to 58 days after PCR confirmation and specificities of 97%, 100%, and 83.8%, respectively. The Abbott assay had 71.3% sensitivity and 100% specificity on the same panel. Parallel or serial algorithms combining two tests did not substantially improve the sensitivity or specificity. Our results showed lower sensitivity and, for one immunoassay, lower specificity compared to the manufacturers’ results and other reported validations. Seroprevalence estimates using these assays might need to be interpreted with caution in Nigeria and similar settings. These findings highlight the importance of in-country validations of SARS-CoV-2 serological assays prior to use to ensure that accurate results are available for public health decision-making to control the COVID-19 pandemic in Africa.

**IMPORTANCE** This study used positive and negative sample panels from Nigeria to test the performance of several commercially available SARS-CoV-2 serological assays. Using these prepandemic and SARS-CoV-2-positive samples, we found much lower levels of sensitivity in four commercially available assays than most assay manufacturer reports and independent evaluations. The use of these assays with suboptimal sensitivity and specificity in Nigeria or countries with population exposure to similar endemic pathogens could lead to a biased estimate of the seroprevalence, over- or underestimating the true disease prevalence, and limit efforts to stop the spread of SARS-CoV-2. It is important to conduct in-country validations of serological SARS-CoV-2 assays prior to their widespread use, especially in countries with limited representation in published assay validations.

## INTRODUCTION

Since Africa reported its first case of coronavirus disease 2019 (COVID-19) on 14 February 2020, scientists have observed that the pandemic has unfolded differently on the continent. Reports from the World Health Organization (WHO) indicate that more than 80% of Africans infected with COVID-19 were asymptomatic, compared to an estimated 40% to 50% seen in the rest of the world; mortality due to COVID-19 was also significantly reduced (1). Asymptomatic cases of infectious diseases complicate the tracking of epidemics and prevent reliable estimates of transmission and thus mitigation measures (2). SARS-CoV-2 serological surveys can be valuable methods to assess asymptomatic infections, monitor SARS-CoV-2 infections across the population, and inform mathematical models that predict the course of the epidemic to guide public health decisions to halt its spread (3).

Validated assays are critical for conducting reliable serological surveys. The use of assays with suboptimal sensitivity and specificity leads to a biased estimate of prevalence. According to WHO, it is desirable that antibody tests for SARS-CoV-2 have at least 98% sensitivity and 99% specificity (4). Several independent studies have validated commercially available serological assays and showed sensitivities of >90% in samples taken at least 14 days after the onset of symptoms and nearly 100% specificity (5–7). The validation of serological assays in the specific regions where they are being deployed for use is important to ensure that the tests meet a minimum requirement for diagnostic performance in the population targeted for use (8, 9). Some independent validations using samples from the United States and Denmark have shown lower levels of sensitivity (10) and specificity (11) for tests, including the Euroimmun immunoglobulin G (IgG) spike protein enzyme-linked immunosorbent assay (ELISA), compared to the manufacturers’ performance characteristics, highlighting the importance of additional test validations. Furthermore, despite a growing number of available SARS-CoV-2 assays, much of the validation data to date comes from populations in China, the United States, and Europe; there is a gap in the validation data of these serological assays in different populations, given especially that the pattern of infection seen in Africa is different from that in the rest of the world (12, 13).

The objective of this study was to assess the analytical performance of five locally accessible commercial serological test kits to detect SARS-CoV-2 antibodies in a Nigerian population, four ELISAs (Euroimmun anti-SARS-CoV-2 nucleocapsid protein [NCP] ELISA [IgG], Euroimmun anti-SARS-CoV-2 spike protein ELISA [IgG], Mologic Omega SARS-CoV-2 IgG ELISA, and Bio-Rad Platelia SARS-CoV-2 Total Ab ELISA) and one chemiluminescent microparticle immunoassay method (Abbott Architect SARS-CoV-2 IgG) that were considered for use in SARS-CoV-2 serosurveys in Nigeria.

## RESULTS

### Samples tested.

Of 100 SARS-CoV-2 PCR-positive samples, 4 were found to have insufficient sample volumes and were excluded from the validation. Similarly, one HBsAg-positive prepandemic sample could not be tested due to insufficient sample volume. Table 1 shows the clinical characteristics of the positive panel. The majority (59.4%) of the SARS-CoV-2-positive samples were taken within 14 days after confirmation of infection. Among 96 SARS-CoV-2-positive samples, 2 (2.1%) samples could not be tested by the Abbott assay due to insufficient sample volume, and 1 (1.0%) could not be tested by the Euroimmun NCP assay. The results of all assays were available for the remaining SARS-CoV-2-positive samples and all 99 prepandemic samples.

**TABLE 1:** Characteristics of SARS-CoV-2 PCR-positive samples

Characteristic^*a*^	*n* (%)^*b*^
Age (yrs)	
Median (IQR)	35.0 (29.5–42.0)
Missing	9 (9.4)
Sex	
Female	48 (50.0)
Male	48 (50.0)
HIV status	
Negative	95 (99.0)
Positive	1 (1.0)
Malaria status, by rapid diagnostic test	
Negative	73 (76.0)
Missing	23 (24.0)
Symptoms	
Asymptomatic	55 (57.3)
Symptomatic^*c*^	34 (35.4)
Missing	7 (7.3)
Days post-confirmation of SARS-CoV-2 infection	
Median (IQR)	13.5 (7–23.25)
0–3	10 (10.4)
4–7	19 (19.8)
8–14	28 (29.2)
15–28	19 (19.8)
29–58	20 (20.8)

a IQR, interquartile range.

b Total *N* = 96.

c Self-report of any of fever, sore throat, runny nose, cough, shortness of breath, vomiting, nausea, and diarrhea.

### Performance characteristics.

**FIG 1:**
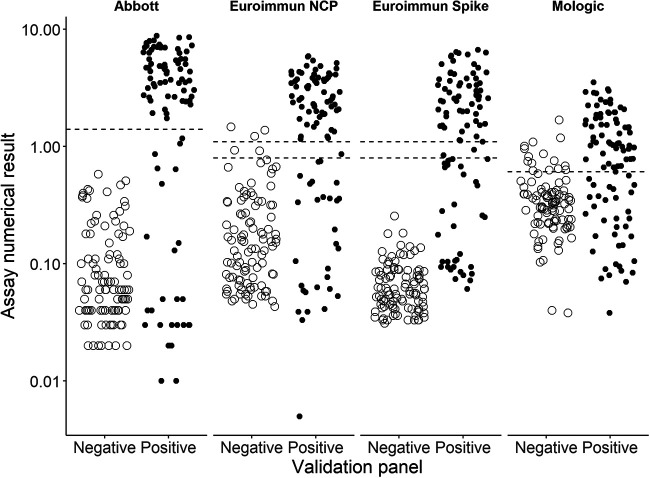
Distribution of numerical results for each assay. The dashed line indicates the assay thresholds. For the Mologic assay, the cutoff was calculated per run; the dashed line represents a mean value for all the cutoff values. The Euroimmun spike and Euroimmun NCP assays have a borderline range as shown between the two dashed lines.

Figure 1 shows the distribution of the numerical assay results for both the positive and negative panels. For the Euroimmun NCP assay, there were two borderline results in the negative panel and one in the positive panel, all of which were negative when retested. On the Euroimmun spike assay, there were two borderline results in the positive panel. One of those samples was negative when retested; the other sample showed a borderline result on the second test and was excluded from the calculations of sensitivity/specificity. The Abbott assay and the Euroimmun spike assay showed the clearest separation of the SARS-CoV-2-positive and -negative samples (Fig. 1), suggesting a higher discriminating power of these tests than others.

The assay sensitivity ranged from 64.2% for the Euroimmun spike assay to 71.3% for the Abbott assay (Table 2). When stratified by the timing of sample collection, the sensitivity was very low, from 10% to 30%, in samples taken ≤3 days post-confirmation of SARS-CoV-2 infection. The sensitivity sharply increased in samples taken between 4 and 7 days postconfirmation and plateaued or increased slightly thereafter (Table 3). The sensitivity estimates of the four assays in samples taken 15 to 58 days post-confirmation of infection ranged from 73.7% to 76.9%. In a multivariable logistic regression model adjusting for days post-confirmation of infection, age, sex, or the presence of symptoms were not associated with false-negative results in any of the test kits.

**TABLE 2:** Overall diagnostic performance of SARS-CoV-2 antibody assays and their combinations^*a*^

Test assay	Sensitivity (% [95% CI])	Specificity (% [95% CI])	Data for 1% prevalence		Data for 5% prevalence	
			PPV (% [95% CI])	NPV (% [95% CI])	PPV (% [95% CI])	NPV (% [95% CI])
Abbott	71.3 (61.0–80.1)	100.0 (96.3–100.0)	100	99.7 (99.6–99.8)	100	98.5 (98.0–98.9)
Euroimmun spike	64.2 (53.7–73.8)	100.0 (96.3–100.0)	100	99.6 (99.5–99.7)	100	98.2 (97.6–98.6)
Euroimmun NCP	68.4 (58.1–77.6)	97.0 (91.4–99.4)	18.6 (6.9–41.2)	99.7 (99.6–99.8)	54.3 (27.9–78.5)	98.3 (97.7–98.7)
Mologic	64.6 (54.2–74.1)	83.8 (75.1–90.5)	3.9 (2.5–6.1)	99.6 (99.4–99.7)	17.4 (11.6–25.2)	97.8 (97.1–98.4)
Parallel^*b*^ (Abbott and Euroimmun spike)	74.2 (64.1–82.7)	100.0 (96.3–100.0)	100	99.7 (99.6–99.8)	100	98.7 (98.1–99.0)
Parallel^*b*^ (Abbott and Euroimmun NCP)	81.7 (72.4–89.0)	97.0 (91.4–99.4)	21.4 (8.2–45.5)	99.8 (99.7–99.9)	58.7 (31.7–81.3)	99.0 (98.5–99.4)
Sequential^*c*^ (Abbott and Euroimmun spike)	61.3 (50.6–71.2)	100.0 (96.3–100.0)	100	99.6 (99.5–99.7)	100	98.0 (97.4–98.4)
Sequential^*c*^ (Abbott and Euroimmun NCP)	58.1 (47.4–68.2)	100.0 (96.3–100.0)	100	99.6 (99.5–99.7)	100	97.8 (97.3–98.3)

a PPV, positive predictive value; NPV, negative predictive value; CI, confidence interval.

b In a parallel strategy, a positive result is defined as either test being positive.

c In a sequential strategy, a sample with a positive result is tested by the second test. A positive result is defined as both tests being positive. The order of the two tests does not change the sensitivity and specificity.

**TABLE 3:** Sensitivity of SARS-CoV-2 antibody assays and their combinations stratified by days post-confirmation of SARS-CoV-2 infection

Test assay	Sensitivity (% [95% CI])^*a*^ for days:							
	0–3	4–7		8–14	15–28	29–58	0–14	15–58
Abbott	20.0 (2.5–55.6)	73.7 (48.8–90.9)		81.5 (61.9–93.7)	77.8 (52.4–93.6)	75.0 (50.9–91.3)	67.9 (54.0–79.7)	76.3 (59.8–88.6)
Euroimmun spike	20.0 (2.5–55.6)	52.6 (28.9–75.6)		75.0 (55.1–89.3)	68.4 (43.4–87.4)	78.9 (54.4–93.9)	57.9 (44.1–70.9)	73.7 (56.9–86.6)
Euroimmun NCP	30.0 (6.7–65.2)	73.7 (48.8–90.9)		70.4 (49.8–86.2)	63.2 (38.4–83.7)	85.0 (62.1–96.8)	64.3 (50.4–76.6)	74.4 (57.9–87.0)
Mologic	10.0 (0.3–44.5)	68.4 (43.4–87.4)		64.3 (44.1–81.4)	73.7 (48.8–90.9)	80.0 (56.3–94.3)	56.1 (42.4–69.3)	76.9 (60.7–88.9)
Parallel^*b*^ (Abbott and Euroimmun spike)	20.0 (2.5–55.6)	78.9 (54.4–93.9)		85.2 (66.3–95.8)	77.8 (52.4–93.6)	78.9 (54.4–93.9)	71.4 (57.8–82.7)	78.4 (61.8–90.2)
Parallel^*b*^ (Abbott and Euroimmun NCP)	40.0 (12.2–73.8)	78.9 (54.4–93.9)		84.6 (65.1–95.6)	88.9 (65.3–98.6)	95.0 (75.1–99.9)	74.5 (61.0–85.3)	92.1 (78.6–98.3)
Sequential^*c*^ (Abbott and Euroimmun spike)	20.0 (2.5–55.6)	47.4 (24.4–71.1)		70.4 (49.8–86.2)	72.2 (46.5–90.3)	73.7 (48.8–90.9)	53.6 (39.7–67.0)	73.0 (55.9–86.2)
Sequential^*c*^ (Abbott and Euroimmun NCP)	10.0 (0.3–44.5)	68.4 (43.4–87.4)		69.2 (48.2–85.7)	50.0 (26.0–74.0)	65.0 (40.8–84.6)	58.2 (44.1–71.3)	57.9 (40.8–73.7)

a CI, confidence interval.

b In a parallel strategy, a positive result is defined as either test being positive.

c In a sequential strategy, a sample with a positive result is tested by the second test. A positive result is defined as both tests being positive. The order of the two tests does not change the sensitivity and specificity.

The specificity was 100% for the Abbott and Euroimmun spike assays, with the Euroimmun NCP assay slightly lower at 97%. The Mologic assay had a significantly lower specificity at 83.8% (95% CI, 75.1 to 90.5) (Table 2). In the negative panel, the frequency of false-positive results did not differ significantly by sample type (HIV positive versus HBsAg positive) in either the Euroimmun NCP kit (2.0% versus 4.1%, *P* = 0.99) or the Mologic kit (14.0% versus 18.4%, *P* = 0.75). When evaluating diagnostic algorithms including two assays, combining the Abbott and the Euroimmun NCP assays in parallel resulted in the highest sensitivity (81.7%; 95% CI, 72.4 to 89.0), while a sequential strategy combining the Abbott and Euroimmun NCP assays resulted in the lowest sensitivity (58.1%; 95% CI, 47.4 to 68.2). There was no loss of specificity for either algorithm.

### Potential impact of using tests in serosurveys.

**FIG 2:**
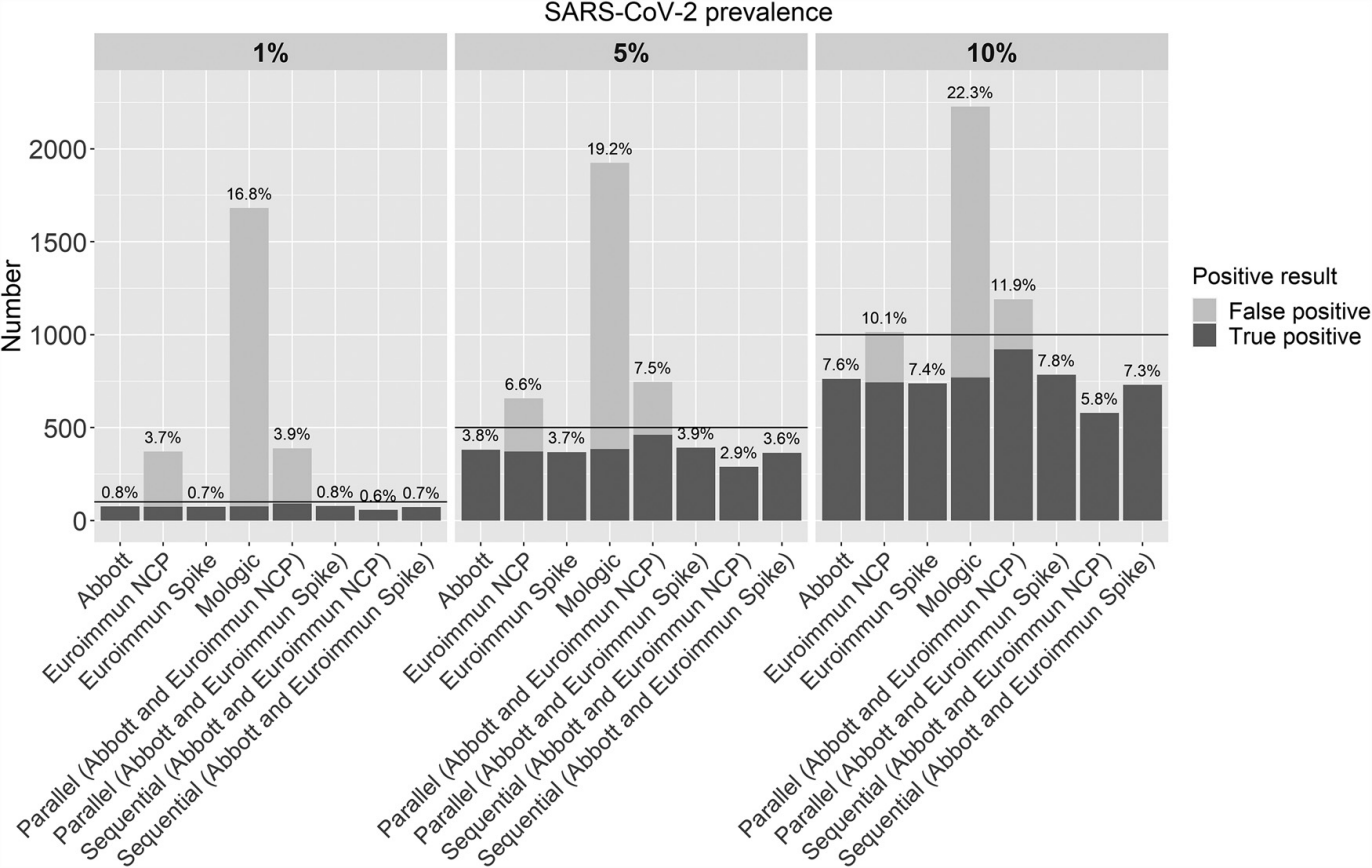
Number of true positives and false positives after testing in 10,000 individuals living in areas with different levels of SARS-CoV-2 prevalence. The horizontal line indicates the true number of people infected with SARS-CoV-2. The percentages indicate the estimated seroprevalence of SARS-CoV-2. A sensitivity estimate for each test in samples >15 days post-confirmation of infection was used for this analysis.

Figure 2 shows the number of positive results when each of these assays was tested in 10,000 individuals under different prevalence settings. Regardless of the background prevalence of SARS-CoV-2, the use of the Omega assay would significantly overestimate the number of infected individuals due to a large number of false-positive results. Likewise, the use of the Euroimmune NCP assay would overestimate the seroprevalence but less so than with the Mologic assay. The Abbott and the Euroimmun spike assays would underestimate the true seroprevalence because of their imperfect sensitivities and specificities of 100%. The performance of the tests alone and in a two-test algorithm depended on the background SARS-CoV-2 seroprevalence; a parallel algorithm using the Abbott and Euroimmun spike assays appeared to perform best, although either assay alone was nearly as good. However, even small changes in the specificity lead to a large increase in the estimated seroprevalence.

## DISCUSSION

We found that the assays tested in this study did not perform as expected when using samples from a Nigerian population, which could have had an impact on public health decision-making if this validation had not been conducted. This study is one of the first validations of SARS-CoV-2 serological assays conducted on samples from an African population, adding to the small but growing evidence pool from the region (12, 13). The assays tested were chosen based on manufacturer-reported performance and availability in Nigeria for use in large household COVID-19 serosurveys. Regarding the two assays with FDA EUA (Abbott Architect and Euroimmun spike), sensitivity for the Abbott was substantially lower than that reported by the manufacturer and other evaluations. Our study found a 76.3% sensitivity of the Abbott assay on day 15 or greater, compared to 100% sensitivity on day 14 or greater according to Abbott (14) and one independent evaluation (15) and 95.2% for samples from patients 12 or more days from symptom onset according to another evaluation (16). For the Euroimmun spike assay, the sensitivity according to the manufacturer at 11 or more days after PCR test is 81% (and according to an independent evaluation, 90%—the timing of samples post-symptom onset or PCR test not available) (17) and thus comparable to the sensitivity we found. Other independent validations have found sensitivities for the Euroimmun spike assay of 78% at day 21 or more after PCR confirmation (10) and of 100% at day 4 or more (5).

Independent evaluations of the Mologic IgG ELISA and the Euroimmun NCP assay are more limited but showed higher sensitivities than our validation. A semi-independent evaluation of the Mologic IgG ELISA found a markedly higher sensitivity than we did, at 94% (95% CI, 89.6% to 96.8%) for samples on day 10 or more post-laboratory diagnosis (18). One previous evaluation of the Euroimmun NCP assay found sensitivities of 88.9% for samples (and 92.9% for the same samples using the Euroimmun spike assay) collected 26 or more days after a positive SARS-CoV-2 PCR (19), and the sensitivity according to the manufacturer is 86.7% for samples collected more than 10 days after symptom onset for PCR test (20). The lower sensitivities observed in our study might be explained by differences in the severity of disease. Several studies have suggested higher levels of SARS-CoV-2 antibody in patients with severe versus mild disease (21–23). Although we did not find a significant difference in sensitivity correlated with the presence of symptoms, our sample size was relatively small, and the samples were mostly provided from ambulatory patients. We also found that the testing algorithms of the assays included improved performance only marginally over single tests. However, even small changes in test specificity can have large impacts on the false-positivity rate; thus, validation of assay specificity using a larger negative sample set might be warranted.

The assay specificity was comparable or higher for three of the four tests compared to manufacturer reports and previous independent evaluations. We found specificities of 100% on the Abbott test using HIV-positive and HBsAg-positive samples collected in 2019 in Nigeria, compared to manufacturer-reported specificities of 99.6% for panels of pre-COVID-19 samples and samples with other respiratory illnesses (*n* = 1,070) (14) and specificities of 99.6% by one recent evaluation (24) and 100% reported by two additional independent evaluations (15, 16). The reported specificity of the Euroimmun spike assay on U.S., European, and Chinese panels pre-COVID-19 (*n* = 1,445) is 98.7%, according to the manufacturer (17). One evaluation showed a slightly lower specificity of 96% in 82 samples, with some false positives among sera from patients positive for dengue virus, cytomegalovirus, and Epstein-Barr virus (EBV) (10). Another study indicated that 2 of 28 samples from patients with common human coronaviruses (types NL63 and OC43) were weakly cross-reactive with the Euroimmun spike assay (5).

Our validation found a slightly lower specificity (97%) of the Euroimmun NCP assay compared to the 99.8% found by the manufacturer using pre-COVID-19 panels from Germany, the United States, and China, including samples positive for influenza and EBV and rheumatoid factor-positive samples (*n* = 1,140) (25). We found a much lower specificity of the Mologic assay at 83.8%, compared to a semi-independent evaluation with a specificity of 97% (18).

Recent validations of SARS-CoV-2 serological assays using African samples have found much lower specificities than in other populations. A multicountry study found specificities of 89.9% for the Euroimmun NCP assay and 94.9% for the Euroimmun spike assay using samples from Ghana, Madagascar, and Nigeria (*n* = 198), compared to 100% using samples from the Lao People’s Democratic Republic and Germany (*n* = 95); the authors speculated that the cross-reactivity might be due to malaria (12). Another study of pre-COVID-19 samples from febrile patients in Benin found that 8 of 60 samples were positive using the Euroimmun spike assay, and a slightly different set of 8 patients was positive using the Euroimmun NCP assay, with the authors also speculating that malaria was responsible for the cross-reactivity, as there were no differences between the negatives and false positives on responses to common human coronaviruses (13). Since it is likely that the majority of the negative-control panel in our sample came from Lagos residents, who have significantly lower malaria exposure compared to other states in Nigeria (14), it is possible that this sample set is not representative of the high malaria exposure that most Nigerian populations have. A related evaluation by this group found that selected Plasmodium falciparum antibodies were significantly higher among prepandemic samples that were false positive on several SARS-CoV-2 serological assays (26).

It is also possible that other exposures and existing antibodies might be responsible for some of the lower specificity with Euroimmun NCP and the Mologic assays. For example, one recent study using African samples found significant cross-reactivity of common human coronaviruses using pre-COVID-19 samples and SARS-CoV-2 serological assays (27), indicating that further investigation into the potential causes of cross-reactivity is warranted.

## LIMITATIONS

This study had several limitations. Although the positive panel was stratified by time elapsed since positive PCR for SARS-CoV-2, this was not as standardized as time since symptom onset, which might be a more appropriate factor influencing the likely appearance of antibodies. Many COVID-19 patients did not report any symptoms, and symptom onset data were not systematically available for those patients reporting symptoms. For the negative panel, samples from HIV-positive and HBsAg-positive individuals only were used, while a number of other diseases could be responsible for cross-reactivity beyond these two. Notably, malaria antibody levels, which might have some cross-reactivity with SARS-CoV-2 antibodies (12, 13), were not available for these samples. Despite the limitations, this study presents one of the most comprehensive validations to date of multiple commercially available SARS-CoV-2 immunoassays using samples from Nigeria.

## CONCLUSION

The findings from this study highlight the importance of in-country validations of SARS-CoV-2 serological assays prior to widespread use, as well as the need to conduct additional testing on more extensive, well-characterized negative panels from a wider variety of populations to examine potential cross-reactivity issues, to ensure that accurate results are available for public health decision-making to control the COVID-19 pandemic in Africa.

## MATERIALS AND METHODS

### Study design.

Laboratory validation was conducted to determine the sensitivity and specificity of commercially available serology test kits for the detection of IgG antibodies specific to the SARS-CoV-2 virus using positive and negative panels from Nigeria. The validation was conducted within a 6-month period from May to October 2020. The samples included negative prepandemic archived plasma and plasma samples from positive COVID-19 patients diagnosed using PCR-based methods at various time points post-PCR confirmation. We adopted a draft of the African Medical Devices Forum protocol for performance laboratory evaluation of COVID-19 serology assays (supplemental material).

### Validation laboratory.

All laboratory analyses were conducted at the Center for Human Virology and Genomics (CHVG), Nigerian Institute of Medical Research (NIMR), Yaba, Lagos, Nigeria. The center is ISO 15189 accredited and a WHO-prequalified *in vitro* diagnostics reference laboratory.

### Sample selection and panel preparation.

The SARS-CoV-2-negative panel consisted of 100 prepandemic archived plasma samples from the biorepository of the CHVG, NIMR, 50 HIV-positive samples and 50 hepatitis B surface antigen (HBsAg)-positive samples collected and archived before October 2019, prior to the COVID-19 pandemic. HIV and hepatitis B status was confirmed using an ELISA method. The positive panel consisted of convalescent plasma samples collected from ambulatory participants 18 years and older visiting the NIMR modified drive-through center, who had been diagnosed SARS-CoV-2 positive using PCR-based methods at NIMR. The study participants consented to specimen storage and future testing after testing positive for the SARS-CoV-2 virus at NIMR. After giving consent, the patients were invited back for blood draws within 0 to 3 days, 4 to 7 days, 8 to 14 days, 15 to 28 days, and greater than or equal to 29 days. All samples were contributed by single individuals except for two individuals, who contributed positive convalescent-phase samples at two different time intervals. Both the archived prepandemic plasma samples and the plasma samples from the positive patients were stored at −20°C in the NIMR biorepository with no dilution prior to storage and a maximum of three freeze/thaw cycles.

### SARS-CoV-2 PCR-based testing.

Two different methods were used for molecular testing: the Cobas SARS-CoV-2 test on the Cobas 6800 system (Roche Diagnostics, Basel, Switzerland) and the BGI Group (BGI) real-time fluorescent reverse transcription-PCR (RT-PCR) kit for detecting SARS-CoV-2. The Cobas 6800 system is a fully automated system for sample-to-result qualitative detection of SARS-CoV-2. The Cobas targets the conserved regions within the ORF1ab gene and the E gene. The procedure was carried out according to the manufacturer’s instructions. For the BGI real-time fluorescent RT-PCR, viral RNA was extracted from oropharyngeal and nasal swabs using the QIAamp viral RNA minikit (Qiagen, Hilden, Germany) and then tested using the BGI real-time fluorescent RT-PCR kit to detect SARS-CoV-2. The BGI assay targets the SARS-CoV-2 ORF1 region. The viral extraction and BGI assay were conducted following the manufacturers’ instructions.

### Antibody tests.

*Euroimmun anti-SARS-CoV-2 ELISA (IgG) (Euroimmun, Lübeck, Germany).* This assay is a semiquantitative ELISA for the detection of IgG antibodies against SARS-CoV-2 spike protein subunit 1 (S1) in human serum or plasma. The assay results are expressed as a ratio, which is calculated by dividing the optical densities (OD) of the sample by those of an internal calibrator provided with the test kit. The cutoffs for interpretation of the results were based on the manufacturer’s recommendations: positive, ≥1.1; borderline, ≥0.8 to <1.1; and negative, <0.8.

*Euroimmun anti-SARS-CoV-2 NCP ELISA (IgG) (Euroimmun, Lübeck, Germany).* This assay is a semiquantitative ELISA for the detection of IgG antibodies against SARS-CoV-2 nucleocapsid protein (NCP) in human serum or plasma. The test was interpreted as noted above for the Euroimmun spike assay.

*Mologic COVID-19 IgG ELISA kit (Omega Diagnostics Group, PLC, UK).* This kit is a semiquantitative ELISA for the detection of IgG antibodies against SARS-CoV-2 NCP and domain 2 spike protein (S2) antigens in human serum or plasma. The test was interpreted in accordance with the manufacturer’s instructions, where samples with ODs greater than the cutoff control +10% were regarded as positive, while ODs below the cutoff control indicated that the sample was negative.

*Platelia SARS-CoV-2 Total Ab ELISA (Bio-Rad Laboratories, Inc., France).* The Platelia SARS-CoV-2 Total Ab assay is a semiquantitative ELISA in a one-step antigen capture format, for the detection of IgM/IgA/IgG antibodies to SARS-CoV-2 in human serum and plasma specimens, targeting the SARS-CoV-2 NCP. The results were interpreted according to the following specimen ratios: <0.8, negative; between <0.8 and ≥1.0, equivocal; ≥1.0, positive.

*Anti-SARS-CoV-2 IgG assay (Abbott Laboratories, USA).* The automated Abbott Architect Plus i2000sr analyzer and SARS-CoV-2 IgG kit is a two-step qualitative chemiluminescent microparticle immunoassay (CMIA) IgG method for detecting antibodies against the SARS-CoV-2 NCP in human serum or plasma. The Architect Plus i2000sr analyzer calculates the results, and according to the manufacturer’s instructions, a cutoff ratio of 1.4 (specimen/calibrator) is used for interpretation of results, where values of <1.4 are reported as negative and values of ≥1.4 are positive.

The Euroimmun ELISA (IgG) detecting the spike protein, the Abbott Architect CMIA, and the Bio-Rad Platelia assay have all received emergency use authorization (EUA) from the U.S. Food and Drug Administration (28). All five tests were performed according to the manufacturers’ instructions.

The laboratory made eight attempts to validate the Platelia SARS-CoV-2 Total Ab test kit in this study; only two out of eight attempts passed the internal test validation criteria for the assay as specified by the manufacturer, with the negative-control and calibrator values falling outside the stipulated ranges in both directions on six attempts. Troubleshooting sessions were held between the laboratory and the manufacturer, but there was no significant improvement in validating the Platelia SARS-CoV-2 Total Ab assay. Thus, its diagnostic accuracy was not evaluated for this assay.

### Ethical approval.

Ethical approval for this study was obtained from the Nigeria Institute of Medical Research Ethical Review Board with approval number IRB/20/022.

### Statistical analysis.

We estimated the sensitivity and specificity, using the positive and negative panels, respectively, with 95% confidence intervals (CIs) using the exact method. With 100 samples each (positive and negative panel), we estimated that the sensitivity and specificity could be estimated with a margin of error of less than 10% and 5%, assuming a sensitivity of 75% and a specificity of 100%, respectively (i.e., 95% CI, <±10% for sensitivity and ≤5% for specificity). In addition to assessing the diagnostic accuracy of the individual assays, we also tested two types of algorithms combining two different assays: a parallel strategy and a sequential strategy. In the parallel strategy, a positive result was defined as either of the two tests being positive. In the sequential strategy, a positive result was defined as both tests being positive. We included only assays with specificities greater than 95% in the algorithms. We also estimated the sensitivity of the assays and algorithms stratified by days post-confirmation of infection as follows: 0 to 3 days, 4 to 7 days, 8 to 14 days, 15 to 28 days, and 29 to 58 days. The test sensitivity was also estimated by restricting the samples to those taken between days 15 and 58 . We estimated the association between false-negative results and clinical characteristics (age, sex, and symptoms) using a logistic regression model. We fitted models by including one of the above covariates at a time while adjusting for days post-confirmation of SARS-CoV-2 infection. Age was treated as a continuous variable and sex and symptoms as binary variables. We modeled the positive and negative predictive values at SARS-CoV-2 prevalences of 1% and 5%, with 95% confidence intervals (CIs) estimated using a method proposed by Mercaldo et al. (29). We also modeled the number of true-positive and false-positive results in 10,000 individuals within a setting of SARS-CoV-2 prevalences of 1%, 5%, and 10%. For this analysis, we used sensitivities that were estimated in samples taken 15 to 58 days post-confirmation of SARS-CoV-2 infection. All statistical analyses were performed using R version 4.0.2.
