# Supplementary material for: Validation of Commercial SARS-CoV-2 Immunoassays in a Nigerian Population
Source: Microbiol Spectr. 2021 Oct 6;9(2):e00680-21. doi: 10.1128/Spectrum.00680-21 (PMC8510257; doi:10.1128/Spectrum.00680-21)
Supplement: SUPPLEMENTAL FILE 1 — Supplemental material. Download SPECTRUM00680-21_Supp_1_seq2.pdf, PDF file, 0.2 MB [file spectrum00680-21_supp_1_seq2.pdf]

SUPPLEMENTARY MATERIAL

We adopted the African Medical Devices Forum (AMDF) protocol for performance laboratory evaluation of COVID-19 serology assays [Report of Africa Medical Devices Forum COVID-19 Task Force 5 -15 MAY 2020 MAY 2020. https://nepad.org/publication/report-of-africa-medical-devices-forum-covid-19-task-force-5-15-may-2020-may-2020]. The detailed protocol that we have adapted from the AMDF is shown below:

AMDF PROTOCOL FOR LABORATORY VALIDATION OF COVID-19 SEROLOGY ASSAYS:

1. INTRODUCTION

SARS-CoV-2 specific antibodies are part of the immune response to infection, and may be detected during the early, late, convalescent, and post-recovery phases of disease. Detection of a SARS-CoV-2 antibody response may be useful to inform triage and management in cases of active infection, to detect convalescence, and to provide evidence of prior exposure. Currently, WHO does not recommend the use of serology assays and point-of-care for immunodiagnostic tests to establish diagnosis of COVID-19 patients[1],[2]. However, this might change as more evidence of their diagnostic performance is gathered. COVID-19 pandemic has led to rapid development of assays without adequate validation studies to make solid field performance claims. In addition, due to the urgency, most jurisdictions including World Health Organization (WHO) and United States Foods and Drug Administration (US FDA) have done rapid assessments based on desk-top review of the manufacturer's quality management system and listed the assays for Emergency Use Authorisation (EUA).

When there is adequate data from the manufacturer or validated studies by recognized authorities, the role of the laboratories is to conduct limited verification studies using a small

[1] WHO 2020. Laboratory testing for coronavirus disease (COVID-19) in suspected human cases. Interim guidance 19 March 2020.
[2] WHO 2020, Advice on the use of point-of-care immunodiagnostic tests for COVID-19. Scientific brief 8 April 2020.

number of clinical specimens to confirm the manufacture's performance claims. The performance evaluation will determine the accuracy of COVID-19 assays in comparison with established higher-order performance assays like nucleic acid amplification tests (NAAT) such as real-time polymerase chain reaction (RT-PCR) which has been shown to have better performance[3]. Recommended performance characteristics using PCR include sensitivity, specificity, negative and positive predictive values. In addition, a number of operational characteristics will be assessed including the suitability for use in laboratories and/or testing settings with limited infrastructure. The purpose of this protocol is to validate antibody tests to determine immunity. Assays for the detection of COVID-19 antibody are covered in this protocol.

2. Objectives

2.1 Broad objective

The overall objective of the evaluation is to assess the performance of commercially available Enzyme Immuno-Assay (EIA) for the detection of human antibodies specific to SARS-CoV-2 against established performance criteria.

2.2 Specific objectives

The specific objectives of the performance evaluation are:

a) To determine the sensitivity, specificity of commercially available COVID-19 assays EIAs for the detection of COVID-19 antibodies as compared to reference result obtained from RT-PCR.
b) To evaluate agreement between the standard COVID-19 assays EIAs.

3. Approach

3.1 Study design: This is a combination of retrospective and prospective evaluation. We will conduct tests on existing biobanked samples from the pre- COVID era as well as

[3] WHO 2020. Laboratory testing for coronavirus 2019 (COVID-19) in suspected human cases. Interim guidance 2 March 2020.

those collected since the COVID epidemic (collected from well- characterised suspected COVID-19 cases who visit the NIMR drive through center for testing).

3.2 Setting:

The evaluation will be conducted at the Center for Human Virology and Genomics, Microbiology Department, NIMR, a WHO prequalification evaluation reference laboratory with well-trained scientists and/or technologists in settings of well-established quality management systems.

3.3 Study Samples

Plasma or serum specimens will be used to evaluate each of the assays. The sample size will include a minimum of 100 COVID-19 positives and 100 COVID-19 negatives (consisting of 50 HIV and 50 HBV pre-COVID panels). The 100 positive specimens will fall into sub-categories for days from post laboratory confirmation: N=10 for Day 0-3, N=20 for Day 4-7, N = 30 for Day 8-14, N = 20 for Day 15-28, N=20 for Day 29+ in order to cover the antibody spectrum. Confirmation of COVID-19 infection will be based on COVID-19 RT-PCR positive nasal, oropharyngeal (OP) swab or sputum specimen from suspected individuals. Clinical data associated with each sample is available. The identity of the NAT used shall also be documented including the cycle threshold (Ct) value when available.

COVID-19 negative samples will be defined as archived serum or plasma specimens that are unlikely to have any exposure to SARS-CoV-2, specifically 50 HIV and 50 HBV stored specimens collected in 2019 or earlier, prior to the introduction of SARS-CoV-2. Clinical data associated with each sample is available.

Panel specimens will be de-identified and assigned a unique identification number. Once the specimens have been processed and labelled, they shall be aliquoted and frozen immediately at -70°C. During the period of testing, the required number of aliquoted specimens will be stored at 2 - 8 °C and this time period shall not exceed one week. After the completion of testing, they

are again stored at -70°C. The number of freeze-thaw cycles shall be recorded. Each aliquot shall not undergo more than five freeze/thaw cycles, as this has been shown to affect the stability of antibodies causing it to form aggregates and reduction in binding capacity.

3.4 Testing

A standard operating procedure will be prepared for each assay under evaluation. The kits under evaluation will be tested using the plasma or serum specimens according to manufacturer's instructions. Kit controls, if available, will be tested with the kit at the beginning of each test run. The specimens will also be tested on the reference method which is RT-PCR. Each product under evaluation will be used strictly in accordance with the instructions for use (IFU) issued by the manufacturer.

4. Ethical consideration

The evaluations will be performed on specimens in which individuals provided informed consent. All personal identification will be removed before archiving the specimens. The test results obtained from the evaluation shall not be used for patient care.

5. Interpretation of results

Interpretation of results for each assay under evaluation will be made strictly according to the manufacturers' instructions within the IFU. Invalid test results shall be recorded on data collection sheets including where the control line does not appear or in any other way the test result is invalid as defined by the IFU. Visual interpretation of results of subjectively read assays will be made independently by three readers (without the knowledge of the other two sets of results and blinded to the reference result for the specimen) and entered onto the data collection sheets. These results are compared by the operator carrying out the assay so that any mistakes may be identified and rectified immediately. Should recording errors be identified, both the original and corrected result will be recorded and initialed by the reader. When the three readers interpret the results differently from each other (i.e. reactive/non-reactive), the consensus will be recorded as that interpretation which occurs two out of three times.

5.1 Documentation of test results

All test results will be documented on standardized test result worksheets and then entered in a Microsoft Excel spreadsheet for further data analysis. Where applicable for EIAs, test results will be recorded electronically directly from the plate reader and then entered in a Microsoft Excel spreadsheet for further data analysis. Printed records will also be generated from the EIA reader, and these will be used to cross-check a sample of the imported data.

6. Quality Control and interpretation of results

6.1 Test kit controls

Manufacturer-supplied positive and negative test kit controls will be run as indicated in the IFU for all test formats included in each test run for EIAs and at the commencement of each testing session for rapid diagnostic tests and other formats. Where positive and negative test kit controls are not supplied by the manufacturer, as will be the case for many rapid diagnostic tests, the external quality control specimen will act as the control specimen.

7. Data analysis

7.1 Invalid runs/devices

The number of invalid test runs (if EIA) is recorded as the number of invalid runs as a percentage of the total number of runs performed for clinical specimens only.

The number of invalid devices (if rapid diagnostic test or other format) is recorded as the number of invalid test devices as a percentage of the total number of devices used for the entire evaluation.

Invalid results may mean invalid test results as defined by the IFU where the control line/band/spot does not appear or invalid due to obviously defective test device or defective transfer pipette.

7.2 Clinical performance characteristics

Table 1 below shows how to calculate the clinical performance characteristics by comparing the results of the assay under evaluation and the reference testing results on the main clinical specimen panel.

Table 1 – Two by two table for calculation of performance characteristics

	***Reference testing results**			
		SARS CoV-2 positive	SARS CoV-2 negative	Total
**Results of assay under evaluation**	Reactive	a<br>(true positives)	b<br>(false positives)	a + b
	Non-reactive	c<br>(false negatives)	d<br>(true negatives)	c + d
	Total	a + c	b + d	a+b+c+d

*Reference standard RTqPCR.

7.2.1 Sensitivity

Sensitivity is the ability of the assay under evaluation to correctly detect specimens that contain SARS CoV-2 antibodies and/or SARS CoV-2 antigen (reference results positive). Thus, sensitivity is the number of true positive specimens identified by the assay under evaluation as positive (a), divided by the number of specimens identified by the reference assays as positive (a+c), expressed as a percentage.

$$\text{Sensitivity} = \frac{a}{a+c}$$

In addition to the overall analysis, we will stratify results by timing of sample collection.

7.2.2 Specificity

Specificity is the ability of the assay under evaluation to detect correctly specimens that do not contain SARS CoV-2 antibodies and/or SARS CoV-2 antigen (reference results negative). Thus, specificity is the number of true negative specimens identified by the assay under evaluation as negative (d), divided by the number of specimens identified by the reference assays as negative (b+d), expressed as a percentage.

Specificity = $\frac{d}{b+d}$

7.2.3 Confidence intervals

The exact 95% confidence intervals for binomial proportions are calculated for both sensitivity and specificity.

7.3 Initial and final sensitivity and specificity

The initial sensitivity and specificity are calculated based on the initial results obtained for the assay under evaluation (except for invalid results, for which the results of repeated testing is used).

The final sensitivity and specificity values are calculated taking into consideration the repeat testing performed on a same lot of the assay under evaluation.

8. Acceptance criteria

At the time of finalizing this protocol (June 2020), there were no agreed minimum acceptable performance for COVID-19 serology assays (ELISA) decided and published in Nigeria. We have thus decided on a pragmatic approach. At a minimum, this validation study will inform whether the same performance estimates reported by the manufacturers are obtainable. The estimates for specificity as well as sensitivity, stratified by timing of sample collection, will be reviewed and implications discussed by the study investigators to decide on the adoption of the test kits for specific objectives, while taking into account the latest guidance (e.g TPP[4]) from the WHO and

other agencies such as Public Health England. A decision will be made then on which test(s) is used for the seroepidemiology study and, which tests should be used in combination to achieve the required acceptable diagnostic performance.

Sample selection and panel preparation

The SARS-CoV-2-negative panel consisted of 100 pre-pandemic archived plasma samples from the biorepository of Center for Human Virology and Genomics, Nigerian Institute of Medical Research (CHVG, NIMR): 50 HIV-positive samples and 50 hepatitis B surface antigen (HBsAg) positive samples collected and archived before October 2019, prior to the COVID-19 pandemic. The CHVG, NIMR is both an ISO 15189 accredited laboratory and is listed as a WHO prequalification laboratory. CHVG conducts HIV and HBsAg confirmation using ELISA methods which have both been validated. Samples are tested neat, without dilution in accordance to manufacturers' instructions. The laboratory has evaluated the performance of several IVDs in the past. Since 2004, it has been responsible for evaluating HIV rapid test kits for our national regulatory body and for product distributors in the country. The objective of the performance evaluation is to ensure acceptable performance of the test kits before product registration in the country. CHVG has in her biorepository a pool of characterized panels for HIV and hepatitis B. The laboratory is enrolled with the College of American Pathology which is an independent external quality assessment provider to ensure her proficiency. The laboratory has maintained satisfactory performance in these schemes over the years. The laboratory provides routine diagnostic services for HIV-1 confirmation and HBsAg assay, for our institutional HIV treatment center which cares for over 20,000 patients and other health facilities. These are the sources for the continuous access to clinical samples from where panels are prepared for performance evaluation.

CHVG provides routine pathology services and is also a national reference laboratory for HIV and viral hepatitis. Blood samples are collected from NIMR outpatient clinic, all general hospitals and primary health centers in Lagos supported by implementing partners and numerous other private and government hospitals. The laboratory has a databank of over 100,000 samples. As a national

reference laboratory, it receives human samples for HIV, HBsAg and other analytes. These samples are characterized and panels are prepared for storage at -20°C with a maximum of three freeze thaw cycles. From the stock of panels, HIV panels are sent to other testing facilities to assess their proficiency periodically. This has been published and the link is http://dx.doi.org/10.4102/ ajlm.v3i1.102.

On the average, the laboratory receives about 100 patients daily from whom venous whole blood is collected. The blood is processed and the plasma is stored in two aliquots. One aliquot is used for testing and the other vial is kept for long storage. We have method validation reports for all assays including HIV and HBsAg serology. A database of the storage inventory and test results is maintained. There is a validation system in place to ensure the accuracy of stored information. We have adequate personnel and infrastructural facilities for this purpose.
